# Identification of a basement membrane gene signature for predicting prognosis and estimating the tumor immune microenvironment in prostate cancer

**DOI:** 10.18632/aging.205445

**Published:** 2024-01-17

**Authors:** Tao Xie, Du-Jiang Fu, Kang-Jing Li, Jia-Ding Guo, Zhao-Ming Xiao, Zhijie Li, Shan-Chao Zhao

**Affiliations:** 1 Department of Urology, Nanfang Hospital, Southern Medical University, Guangzhou 510515, China; 2 Department of Urology, The Third Affiliated Hospital of Southern Medical University, Guangzhou 510500, China; 3 Department of Geriatric Medicine, Shenzhen People’s Hospital (The Second Clinical Medical College, Jinan University, The First Affiliated Hospital, Southern University of Science and Technology), Shenzhen 518020, China

**Keywords:** prostate cancer, basement membrane gene, recurrence-free survival, tumor immune microenvironment, drug sensitivity

## Abstract

Basement membrane plays an important role in tumor invasion and metastasis, which is closely related to prognosis. However, the prognostic value and biology of basement membrane genes (BMGs) in prostate cancer (PCa) remain unknown. In the TCGA training set, we used differentially expressed gene analysis, protein-protein interaction networks, univariate and multivariate Cox regression, and least absolute shrinkage and selection operator regression to construct a basement membrane-related risk model (BMRM) and validated its effectiveness in the MSKCC validation set. Furthermore, the accurate nomogram was constructed to improve clinical applicability. Patients with PCa were divided into high-risk and low-risk groups according to the optimal cut-off value of the basement membrane-related risk score (BMRS). It was found that BMRS was significantly associated with RFS, T-stage, Gleason score, and tumor microenvironmental characteristics in PCa patients. Further analysis showed that the model grouping was closely related to tumor immune microenvironment characteristics, immune checkpoint inhibitors, and chemotherapeutic drug sensitivity. In this study, we developed a new BMGs-based prognostic model to determine the prognostic value of BMGs in PCa. Finally, we confirmed that THBS2, a key gene in BMRM, may be an important link in the occurrence and progression of PCa. This study provides a novel perspective to assess the prognosis of PCa patients and provides clues for the selection of future personalized treatment regimens.

## INTRODUCTION

Prostate cancer (PCa) is a worldwide disease that affects men’s health and has risen to become the second most common malignancy in men [1]. In recent years, the incidence and mortality rate of PCa in China have also been increasing. Radical prostatectomy, external radiation therapy, and brachytherapy are the recommended interventions for localized PCa [2]. However, tumor recurrence is a clinical dilemma in the management of PCa, especially in patients with high tumor grade and high tumor stage [3]. It is estimated that > 40% of men with intermediate or high-risk PCa will experience biochemical recurrence after radical prostatectomy [4]. Therefore, it is important to find reliable biomarkers to intervene in patients with a high risk of recurrence to guide treatment strategies and prognostic assessment.

The basement membrane is an extracellular matrix (ECM) composed mainly of laminin and type IV collagen that serves as a structural barrier to tumor invasion and metastasis [5]. Underscoring their diverse and essential functions, variants in more than 20 basement membrane genes (BMGs) underlie human diseases [6]. BMGs may be predominantly expressed in stromal cells, particularly in cells involved in the production and maintenance of the basement membrane, such as fibroblasts and myoepithelial cells. These cells synthesize and secrete matrix components, forming the structure of the basement membrane [7, 8]. However, Laminin-511 has been implicated in cell migration, tumor growth, and metastasis, with higher levels of this isoform found in many breast, lung, thyroid, and prostate cancers [9, 10]. Tumor cells must penetrate the basement membrane during metastasis. Therefore, degradation of the basement membrane and ECM is a prerequisite for cancer invasion and distant metastasis [7]. PCa cells interact with the ECM to adjust its growth and metastasis [11]. Disruption of basement membrane continuity as well as the synthesis of basement membrane proteins is observed during PCa progression [12]. Fuchs et al. found that basement membrane staining was reduced in high Gleason grade PCa and completely disappeared in metastases [13]. It has been found that matrix metalloproteinase-7 (MMP-7) disrupts and degrades the perlecan complex bound in the ECM, thereby facilitating circulating tumor cell production and distant metastasis in PCa [14].

Furthermore, as a specialized structure of the ECM, the basement membrane participates in the composition of the tumor immune microenvironment (TIME) [7]. As the target of autoantibodies in immune diseases, BMGs are believed to regulate tumor immunity in addition to affecting tumor cell proliferation, invasion and migration [15]. It has been reported that laminin not only regulates T-cell adhesion and migration, but also directly correlates with patient prognosis and PD-1/PD-L1 treatment response [16]. For these reasons, BMGs as tumor ECM components are considered as therapeutic targets for cancer.

The aim of this study was to investigate the role of BMGs in the early prediction of biochemical recurrence of PCa and in guiding treatment decisions. Based on five differentially expressed BMGs, we constructed a prognostic model to predict recurrence-free survival (RFS) in PCa patients and found a strong correlation between BMGs and TIME. Our findings will provide guidance for the development of personalized treatment protocols to improve the prognosis of PCa patients.

## MATERIALS AND METHODS

### Data acquisition and processing

The mRNA expression data and corresponding clinical information for 501 PCa and 52 adjacent normal samples were obtained from The Cancer Genome Atlas (TCGA, https://portal.gdc.cancer.gov) database. Ultimately, patients containing clinical information such as age, pathological T-stage, prostate-specific antigen (PSA), Gleason score, and time to recurrence (n = 423) were screened as a training cohort for the subsequent study. Similarly, 140 PCa patients from the Memorial Sloan Kettering Cancer Center (MSKCC) cohort (GSE21034, https://www.ncbi.nlm.nih.gov/geo/) were retained by the above screening criteria and used as an external validation cohort. 224 BMGs were obtained from previous studies [17] (Supplementary Table 1). The clinicopathological characteristics of patients in the TCGA-PRAD cohort and MSKCC cohort are detailed in Supplementary Table 2.

### Identification and functional enrichment analysis of DE-BMGs

Differentially expressed genes (DEGs) were calculated in the TCGA-PRAD dataset using the “Limma” R package [18]. 120 differentially expressed BMGs (DE-BMGs) were included in the PCa based on a cut-off value of fold change > 1.5 and FDR < 0.05. The DE-BMGs are shown by a volcano plot and heat map. Subsequently, Gene Ontology (GO) and Kyoto Encyclopedia of Genes and Genomes (KEGG) enrichment analyses were performed using the “ClusterProfiler” R software package [19] to investigate the biological functions of the DE-BMGs.

### Identification of prognosis-associated BMGs and their protein-protein interaction (PPI) network and copy number variation (CNV) landscapes

We performed univariate Cox regression survival analysis using the “survival” R package to extract DE-BMGs associated with RFS for further study. Subsequently, the mRNA expression of the above-mentioned genes was analyzed differentially in the TCGA-PRAD data. The PPI network of prognosis-related DE-BMGs was assessed using the Search Tool for the Retrieval of Interaction Genes (STRING, https://STRING-db.org). Amplifications and deletions of DE-BMGs were identified based on CNV data, and the CNV landscape was visualized using the R software package “OmicCircos” [20].

### Construction and validation of a prognostic model based on BMGs

Following univariate Cox regression analysis with the least absolute shrinkage and selection operator (LASSO), five stable prognostic BMGs were identified, and further prognostic basement membrane-related risk models (BMRM) were constructed [21]. Regression coefficients for genes in the TCGA-PRAD cohort were obtained by multifactorial Cox regression analysis. The basement membrane-related risk score (BMRS) formula was calculated from this regression coefficient as follows: RiskScore=Σexp(BMGs)×coefficient, where coefficient indicates the regression coefficient of the prognostic gene signature. Patients were divided into low- and high-risk groups based on the best cutoff level. The value of BMRM for predicting RFS in PCa patients was assessed by Kaplan-Meier survival curves and time-dependent receiver operating characteristic (ROC) curves. In addition, the MSKCC cohort was selected as an external validation cohort to test the predictive power of prognosis-related BMRM.

### Identification of independent prognostic factors and construction of a nomogram for predicting RFS

Univariate and multivariate Cox regression analyses were performed on the BMRM and clinicopathological parameters of patients in the TCGA-PRAD cohort to identify whether the BMRS could be used as an independent prognostic factor. Next, we combined the BMRS and clinical data to produce a nomogram for RFS [22]. In addition, RFS calibration curves were generated at 1, 3, and 5 years to verify the accuracy of the nomogram. Finally, the correlation between BMRS and clinicopathological features was represented by box plots.

### Analysis of BMRM in relation to PCa patient immune characteristics, TMB and drug sensitivity

To explore the relationship between BMRS and immune cell infiltration, the ESTIMATE algorithm [23] was used to analyze the stromal score, immune score, and ESTIMATE score for each PCa sample. The relative proportions of 22 tumor-specific immune infiltrating cells in the different risk groups of PCa patients were obtained by using the CIBERSORT algorithm [24]. Next, the correlation between BMRS and immune checkpoints was calculated using the Spearman correlation method. Tumor immune dysfunction and exclusion (TIDE) predicts the rate of response to immunotherapy in patients. The TIDE scores were mainly done through the TIDE website (http://TIDE.dfci.harvard.edu/). Subsequently, information on mutations was obtained from the TCGA database, and the tumor mutation burden (TMB) of PCa patients was calculated using the “Maftools” package [25]. Correlations between TMB scores and different risk groups were assessed. Then, the 10 genes with the highest mutation frequency were selected for visualization. In addition, based on the Genomics of Drug Sensitivity in Cancer (GDSC, https://www.cancerrxgene.org/), we used the “pRRophetic” R package [26] to obtain IC50 estimates for specific chemotherapeutic drug treatments to predict drug sensitivity in different risk groups of PCa patients.

### Cell culture and transfection

The human PCa cell lines C4-2, C4-2B, DU145, PC-3 and normal prostate epithelial cells RWPE-1 were purchased from the Cell Bank of the Chinese Academy of Sciences. All cells were incubated at 37° C in a humidified atmosphere with 5% CO2. Cells were cultured in RPMI-1640 medium (Gibco, Waltham, MA, USA) supplemented with 10% fetal bovine serum (FBS, Gibco, Australia) and 1% penicillin/streptomycin (Gibco). For the transient knockdown of target genes, we transfected cells with Lipofectamine 3000 (Invitrogen, Carlsbad, CA, USA) and siRNAs targeting THBS2 according to the manufacturer's instructions. Cells were collected 36 hours after transfection. All siRNAs were synthesized by RiboBio Company (Guangzhou, China). siRNA sequences are shown in Supplementary Table 3.

### Western blotting

Western blotting experiments were carried out as previously described [27]. Primary antibodies used were as follows: THBS2 (ab112543, Abcam, UK), GAPDH (60004-1-Ig, Proteintech, China), and α-Tubulin (66031-1-Ig, Proteintech).

### Cell counting kit-8 (CCK-8) and colony formation assays

CCK-8 and colony formation assays were conducted to measure the PCa cells’ proliferation capacity. Treated cells were placed in a 96-well plate at a density of 2000 cells/well. The cells were cultured in the presence of 100 μL of CCK-8 (1:10 dilution, CK-04, Dojindo, Japan) solution for 2 h at 37° C. Then, the absorbance values of the wells at 450 nm were evaluated using an automatic spectrometer (Thermo Fisher Scientific, Waltham, MA, USA). This procedure was repeated at 1, 2, 3, 4, and 5 days after cell seeding. For the colony formation assay, approximately 1000 cells/well were plated into a six-well plate and incubated for 10-14 days. When colonies appeared, cells were fixed with 4% paraformaldehyde for 5 min and stained for 10 with Giemsa (Solarbio, Beijing, China). All experiments were conducted in triplicate independently.

### Transwell migration assay

The transwell migration assay was performed using transwell chambers (Corning Inc., Corning, NY, USA). Cells were resuspended in serum-free medium and inoculated in the upper chamber (5 × 10^4^ cells per well); RPMI-1640 containing 10% FBS was usually added to the lower chamber. The culture plate was incubated at 37° C for 48 h. After 48 h inoculation, migrated cells were fixed with 4% paraformaldehyde for 5 min, followed by staining for 10 min with Giemsa (Solarbio, Beijing, China).

### Statistical analysis

All statistical analyses were performed in R 4.2.1 or GraphPad Prism 9. Differences between the two risk groups were analyzed using the Wilcoxon test or t-test. Spearman's correlation analysis was used to assess the relationship between immune checkpoints and BMRS. Continuous variables were expressed as the mean ± standard deviation (SD). Any hypothesis tests with a *p*-value <0.05 were considered significant and were two-sided.

### Data availability statement

The datasets analyzed for this study can be found in the TCGA (https://portal.gdc.cancer.gov/) and the GEO database (https://www.ncbi.nlm.nih.gov/geo/).

## RESULTS

### 120 genes were identified as DE-BMGs in PCa

**Figure 1:**
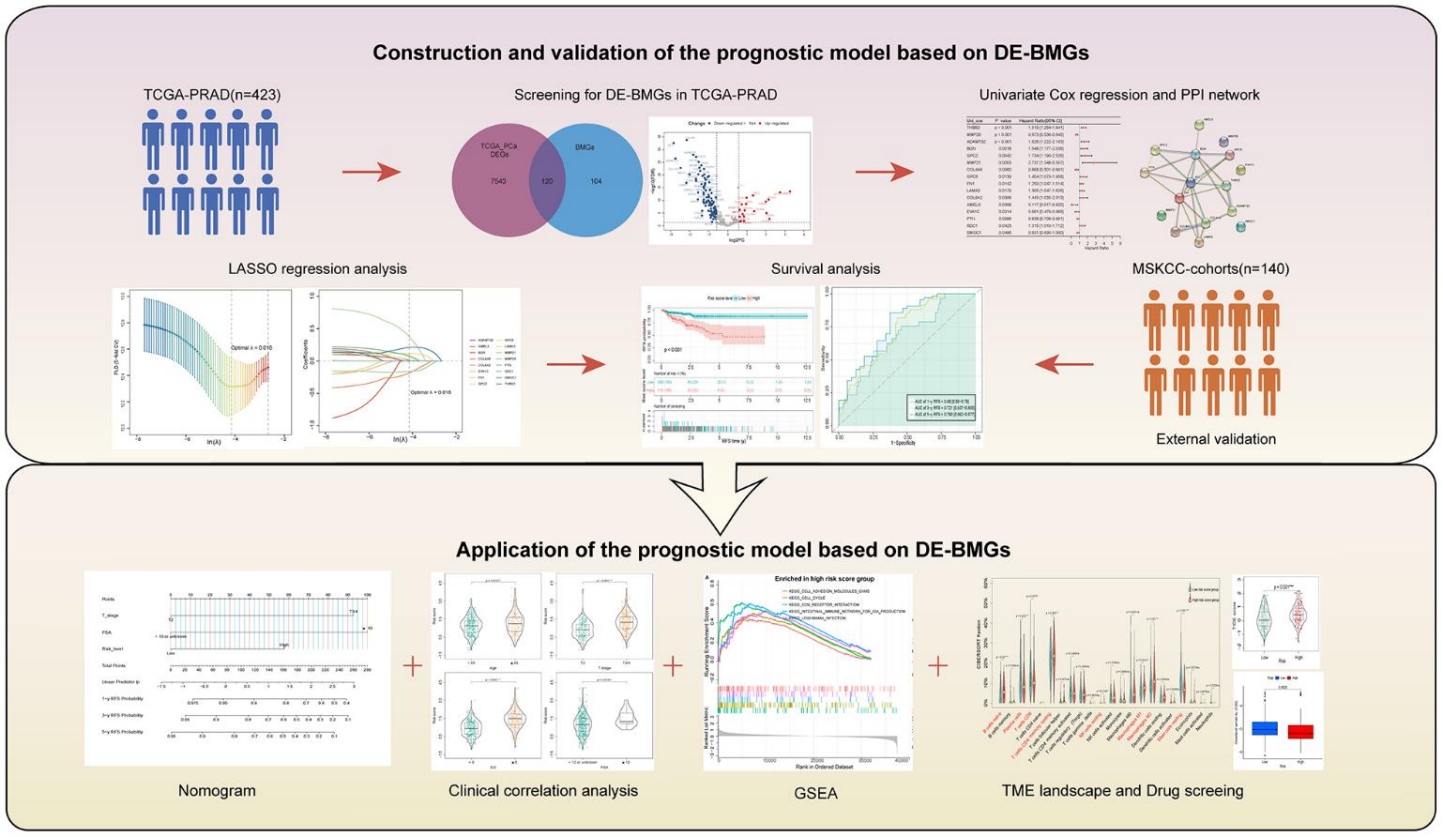
Methodology of the study.

**Figure 2:**
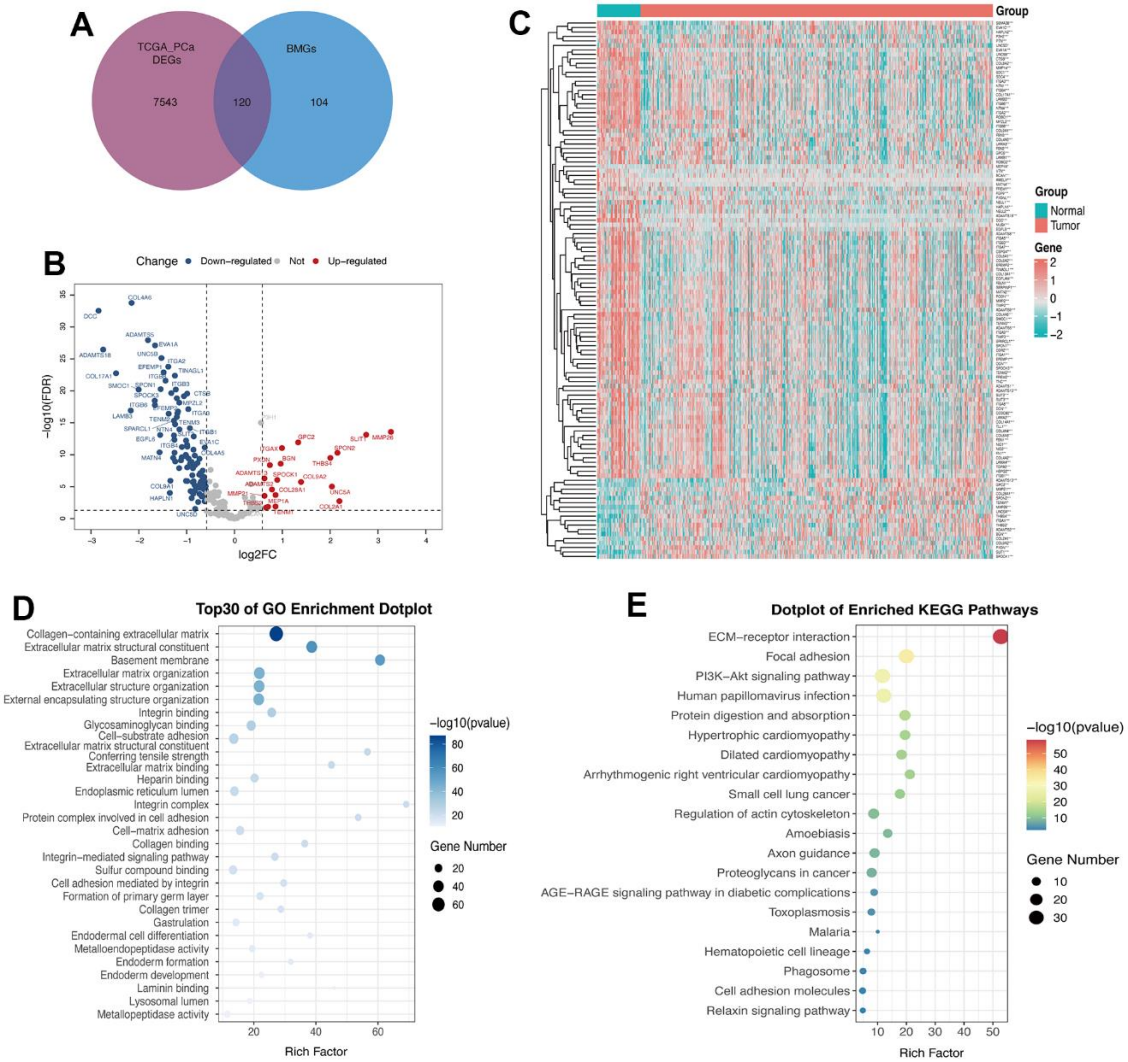
**120 genes were identified as DE-BMGs in PCa.** (**A**) Venn diagram showing the acquisition of DE-BMGs in TCGA-PRAD dataset. (**B**) Volcano map showing 120 DE-BMGs of TCGA-PRAD dataset. Red indicates high expression, blue indicates low expression, and gray indicates no statistically significant difference. (**C**) Heat map of DE-BMGs in PCa (n = 423) and normal prostate tissue (n = 52), where red represents positive correlation and green represents negative correlation. (**D**) Bubble graph of the top 30 terms of DE-BMGs by GO analysis. (**E**) Bubble chart of the top 20 terms of DE-BMGs in KEGG enrichment analysis.

Figure 1 illustrates the methodology of the study. To examine the tumor BMGs landscape in PCa, we performed differential expression analysis in the TCGA-PRAD cohort using the limma package in R. Setting adjusted *p*-values < 0.05 and |FC| > 1.5, a total of 7663 DEGs were identified between the PCa and normal groups. Further, combined with the set of known BMGs genes, the Venn diagram shows that 120 of these DEGs were identified as DE-BMGs (Figure 2A). Compared to normal tissues, 19 genes were upregulated and 101 genes were downregulated in PCa, as shown in the volcano map and heat map (Figure 2B, 2C).

We then implemented GO and KEGG enrichment analyses to understand the potential function of these genes. The terminology of GO enrichment analysis is mainly related to the basement membrane, collagen-containing ECM and ECM structural constituent (Figure 2D). KEGG enrichment analysis showed that DE-BMGs interacted with ECM receptor interaction, focal adhesion and the PI3K-Akt signaling pathway (Figure 2E).

### Identification of DE-BMGs associated with RFS in PCa

**Figure 3:**
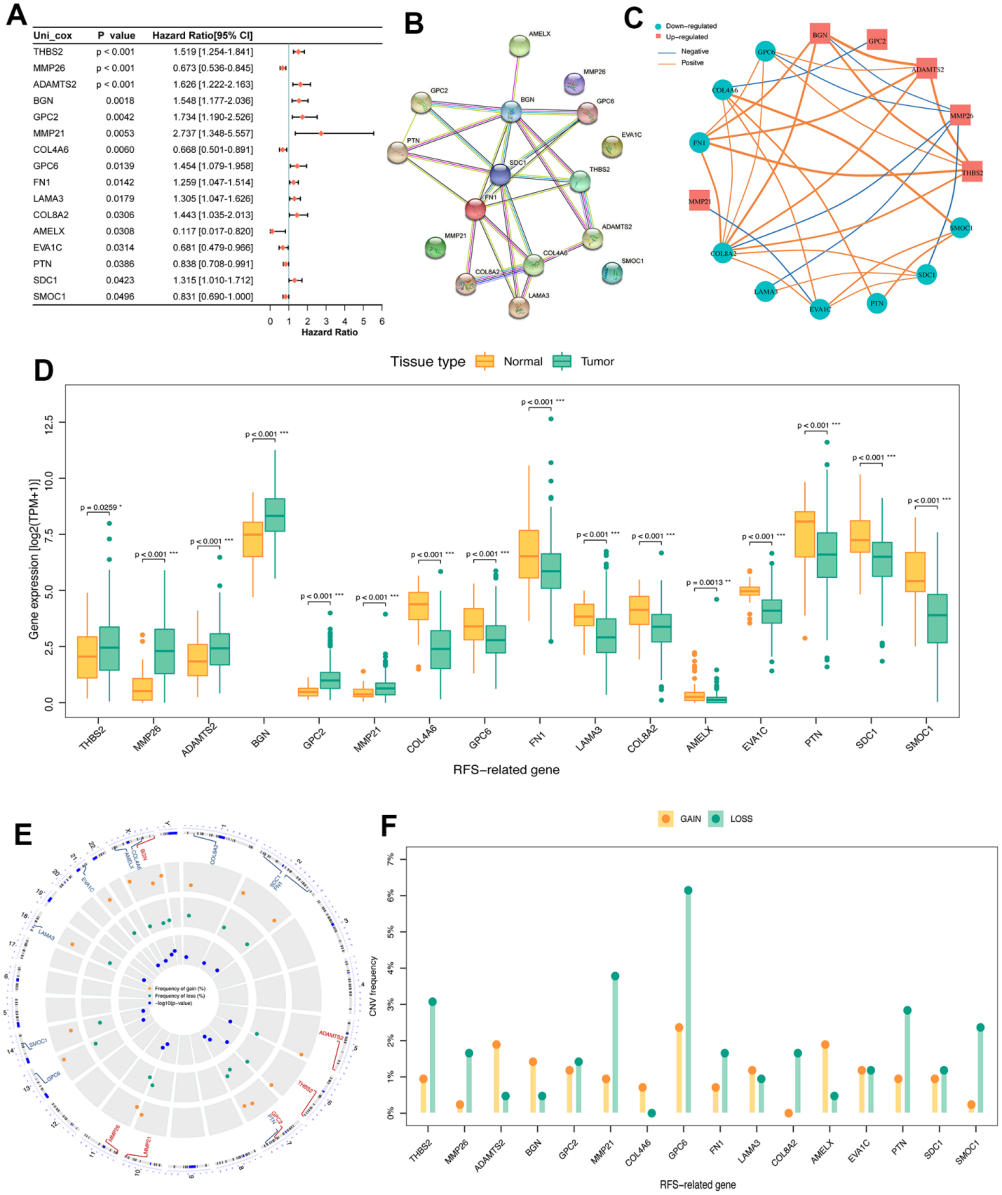
**Identification of prognosis related DE-BMGs in PCa.** (**A**) Forest map of 16 RFS related DE-BMGs in TCGA-PRAD dataset. (**B**) PPI network of 16 RFS related DE-BMGs. (**C**) Co-expression network of 16 RFS related DE-BMGs. (**D**) Expression of 16 RFS related DE-BMGs in PCa and normal tissues. (**E**, **F**) Chromosome location and CNV alteration frequency of 16 RFS related DE-BMGs. Copy number amplification, yellow dot; Copy number deletion, green dot. RFS: Recurrence free survival. *p < 0.05, ** *p* < 0.01, *** *p* < 0.001.

In order to explore the prognostic value of DE-BMGs, we analyzed 120 DE-BMGs obtained from the above TCGA-PCa cohort with univariate Cox analysis and found 16 genes related to RFS. Among them, SMOC1, PTN, EVA1C, AMELX, COL4A6 and MMP26 were protective factors (hazard ratio, HR < 1), while SDC1, COL8A2, LAMA3, FN1, GPC6, MMP21, GPC2, BGN, ADAMTS2 and THBS2 (HR > 1) were risk factors in PCa (Figure 3A). The PPI network showed the complex relationship between these prognostic indicators in PCa, where SDC1, BGN, FN1 and COL4A6 belong to the hub genes of the network (Figure 3B). Moreover, these prognostic indicators were strongly correlated, such as ADAMTS2 and BGN, THBS2 and FN1, COL4A6 and SMOC1 (Figure 3C). Compared with normal prostate tissues, only six genes (THBS2, MMP26, ADAMTS2, BGN, MMP21, and GPC2) were up-regulated in PCa (Figure 3D). Then, we analyzed the CNV landscape of these 16 RFS-related DE-BMGs in PCa. The CNV positions of these 16 genes on the chromosomes were shown in Figure 3E. Among them, BGN, AMELX, ADAMTS2, COL4A6, and LAMA3 exhibited a trend of copy number gains, while SMOC1, PTN, MMP26, SDC1, COL8A2, FN1, GPC6, MMP21, GPC2 and THBS2 showed a trend of copy number losses. In addition, EVA1C was in the neutral group with no copy number change (Figure 3F).

### Establish a RFS-related risk prediction model based on DE-BMGs in PCa

**Figure 4:**
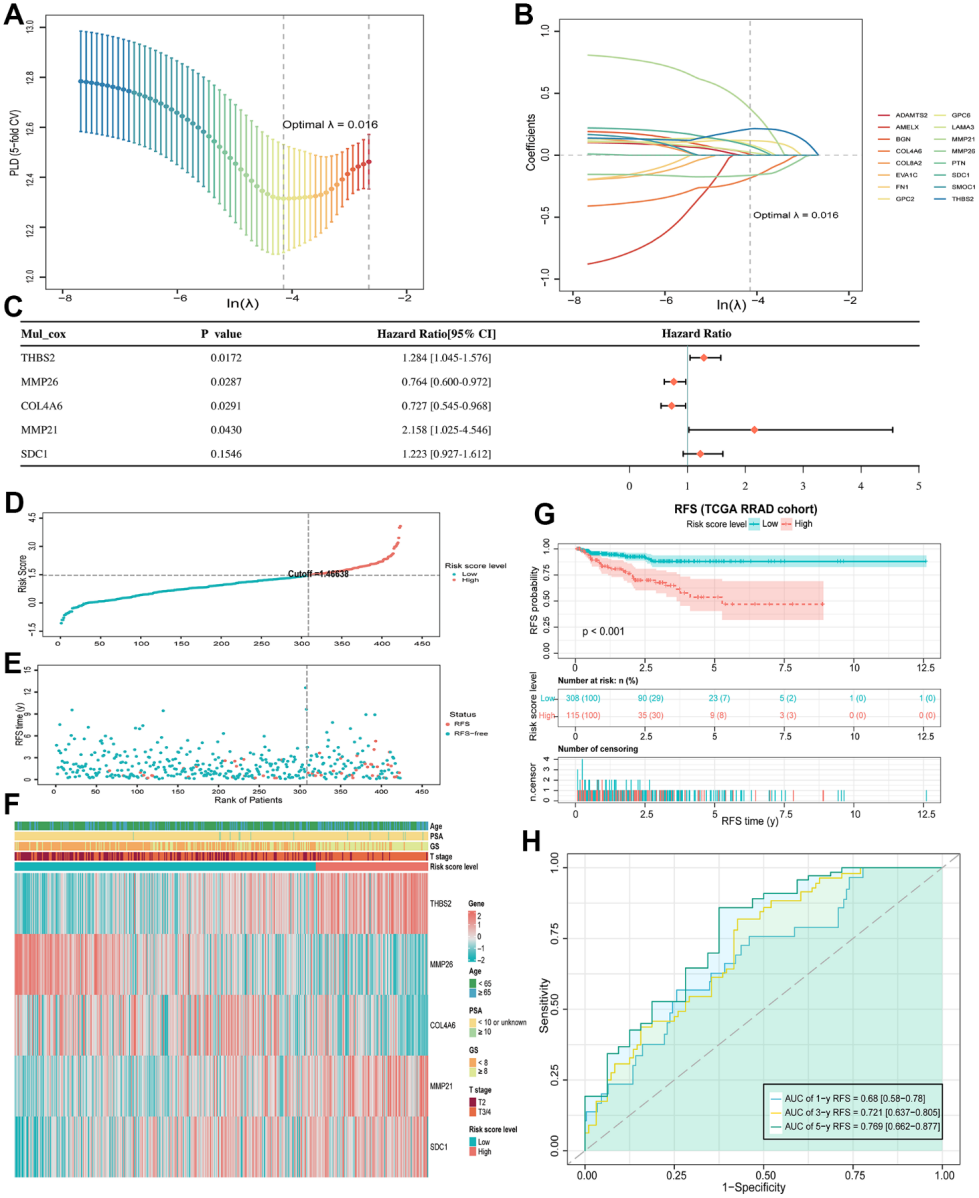
**BMRM construction and prognostic analysis.** (**A**) Selection of the optimal penalty parameter (λ) in the LASSO Cox regression model. (**B**) Gene coefficient spectrum of the 16 RFS-associated BMGs in the TCGA-PRAD cohort in the LASSO Cox regression analysis. (**C**) Forest plot of multivariate analysis showing 5 genes (THBS2, MMP26, COL4A6, MMP21 and SDC1) as independent prognostic factors for RFS in PCa patients. (**D**, **E**) BMRS plots from the TCGA training set. (**F**) Heat map of the two BMRS groups and correlation analysis of clinicopathological characteristics. (**G**) Kaplan-Meier analysis of RFS for PCa patients in both risk groups. (**H**) AUC shows the accuracy of BMRM in predicting 1-, 3-, and 5-year RFS in the TCGA training set.

As mentioned above, among 120 DE-BMGs, only 16 genes were associated with the RFS of patients after univariate Cox regression analysis. Then, LASSO regression analysis was carried out to further narrow down the DE-BMGs related to RFS. A model with a minimum wavelength of 0.016 was selected, and nine genes (MMP21, THBS2, COL4A6, MMP26, GPC2, GPC6, SDC1, LAMA3 and BGN) were identified (Figure 4A, 4B). Then, five genes (THBS2, MMP26, COL4A6, MMP21 and SDC1) were identified as independent prognostic factors by stepwise multiple regression analysis (Figure 4C). Finally, we constructed a BMRM based on the above five genes to predict RFS in PCa patients. The BMRM was constructed based on the following formula: BMRS=0.25×THBS2−0.269×MMP26−0.319×COL4A6+0.769×MMP21+0.201×SDC-1. The BMRS of each patient in the TCGA training set was calculated according to the above formula, and 423 PCa patients were divided into a low-risk group (n = 308) and a high-risk group (n = 115) according to the best cutoff value of 1.46638 (Figure 4D, 4E). From the risk heat map, it can be seen that THBS2, MMP21 and SDC1 are more likely to be expressed in high-risk groups, while MMP26 and COL4A6 were opposite (Figure 4F). Kaplan-Meier survival analysis showed that the RFS time of the high-risk group was shorter than that of the low-risk group (*p* < 0.001; Figure 4G). Besides, the risk model had good prognostic validity in the TCGA training set, and the area under curve (AUC) values of RFS at 1, 3, and 5 years were 0.680, 0.721, and 0.769 respectively (Figure 4H).

### Verify the predictive value of the BMRM in the MSKCC cohort

**Figure 5:**
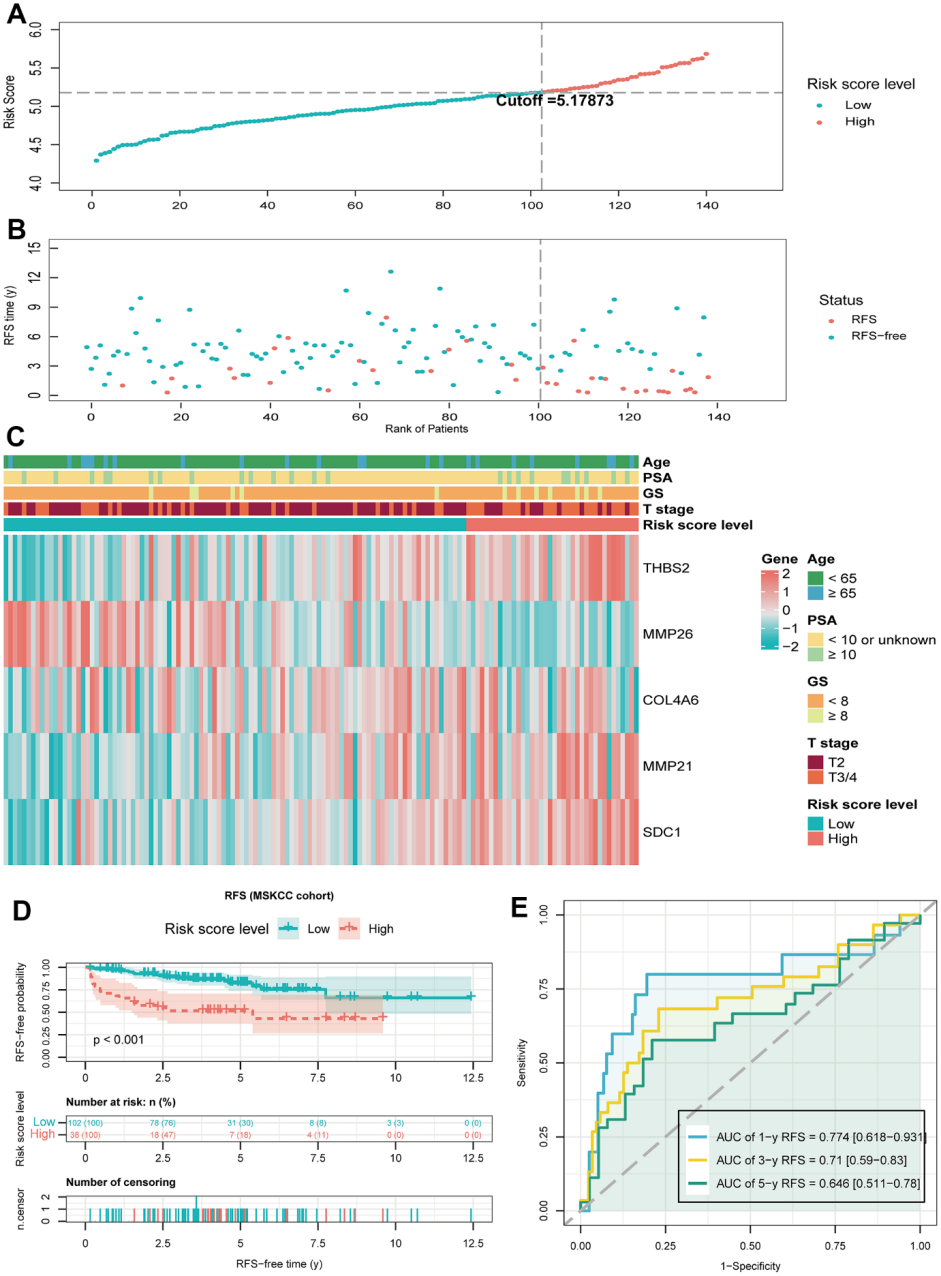
**Validate the predictive performance of BMRM in the MSKCC validation set.** (**A**, **B**) Risk score plots from the MSKCC validation set. (**C**) Risk heat map of the two risk groups in the MSKCC validation set and correlation analysis of clinicopathological characteristics. (**D**) Kaplan-Meier analysis of RFS in the MSKCC validation set for PCa patients in both risk groups. (**E**) AUC shows the accuracy of BMRM in predicting 1-, 3-, and 5-year RFS in the MSKCC validation set.

To further validate the prognostic value of BMRM, we filtered the MSKCC cohort containing RFS information in PCa and used it as an external validation set. Similarly, we used the same analysis in the validation set for the MSKCC cohort as in the TCGA training set, dividing 140 PCa patients into a low-risk group (n = 102) and a high-risk group (n = 38) based on a cutoff value of 5.17873. The distribution of BMRS, patient survival status, and gene expression profiles for the MSKCC cohort are shown in Figure 5A–5C, all of which follow the same trend as the TCGA training set. Kaplan-Meier analysis for the MSKCC cohort also showed a shorter RFS time for the high-risk group, suggesting a worse prognosis (Figure 5D). Similarly, ROC curves were used to assess the prognostic value of BMRM in the MSKCC cohort. Figure 5E demonstrated the AUC values of 0.774, 0.71, and 0.646 for 1, 3, and 5-year RFS in PCa patients, respectively. In summary, we confirmed the significant value of BMRM in predicting the prognosis of PCa patients with the TCGA training set and MSKCC validation set, where five carefully selected DE-BMG genes (THBS2, MMP26, COL4A6, MMP21, and SDC1) may be key molecules in PCa development.

### The relationship between BMRS and pathological and genetic characteristics in PCa patients

**Figure 6:**
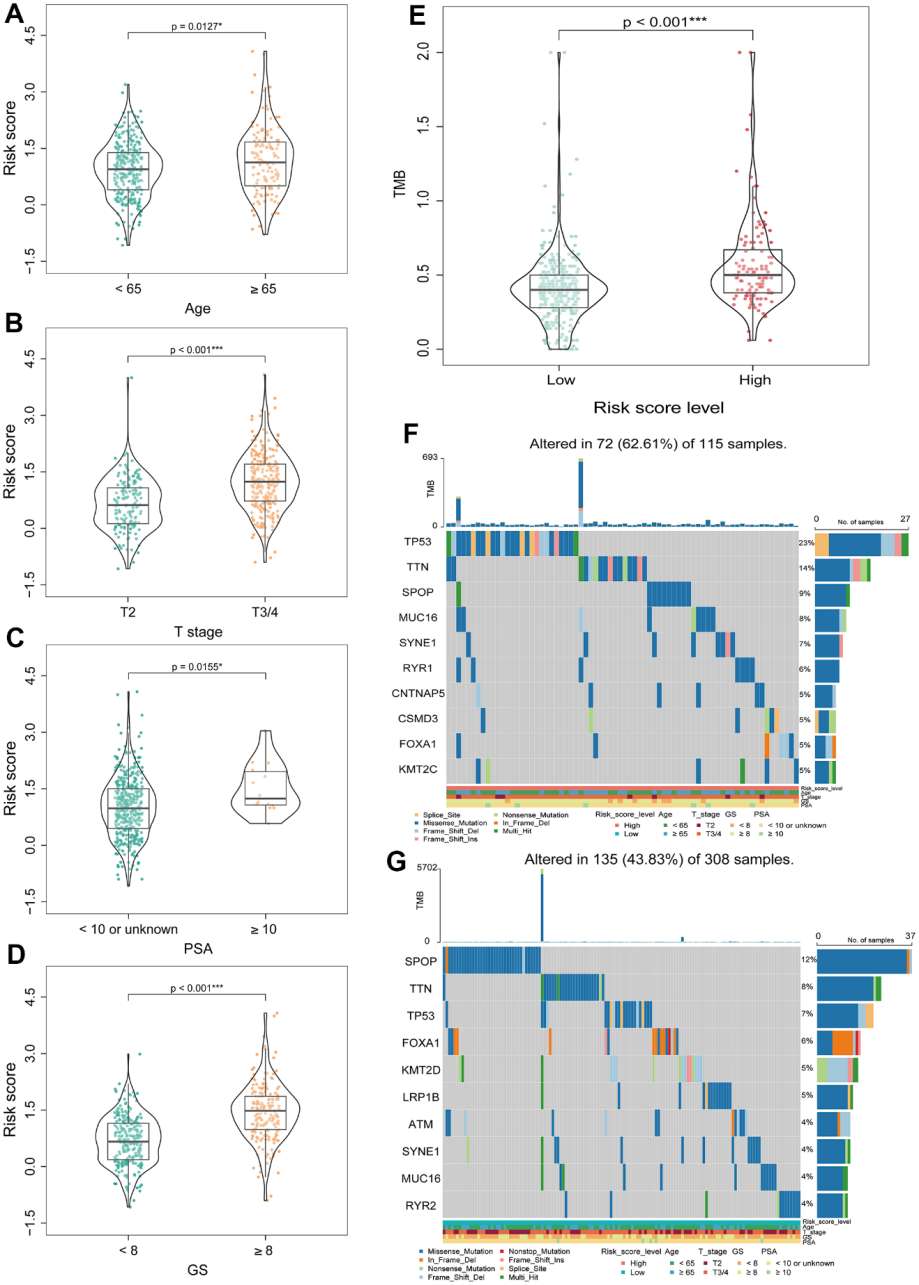
**Correlation analysis between BMRS and clinicopathological characteristics of patients with PCa.** (**A**–**D**) Comparison of differences in BMRS between patients with different Age, T stage, PSA and Gleason score subgroups. (**E**) Analysis of the differences in TMB between the two risk groups. (**F**) Waterfall plot of the top 10 mutated genes in the high-risk group. (**G**) Waterfall plot of the top 10 mutated genes in the low-risk group. * *p* < 0.05, ** *p* < 0.01, *** *p* < 0.001.

Next, we explored the relationship between BMRS and the clinicopathological characteristics of patients with PCa at different stages. The results showed that BMRS in PCa patients was significantly different from age (*p* = 0.0127), T-stage (*p* < 0.001), PSA (*p* = 0.0155) and Gleason score (*p* < 0.001) (Figure 6A–6D). Patients in the high-risk group had significantly increased Gleason score, T-stage and PSA levels. In addition, it was shown that patients with high TMB were more likely to benefit from immune checkpoint inhibitors (ICIs) therapy [28], and we further explored the differences in TMB between the high- and low-risk groups. Surprisingly, the TMB was significantly higher in the high-risk group than in the low-risk group (Figure 6E). In addition, comparing the mutation profiles, the proportion of mutations (like TP53 and TTN) was significantly higher in the high-risk group than in the low-risk group (Figure 6F, 6G).

### Assessment of the independent prognostic value of BMRM and construction of a nomogram in PCa patients

**Figure 7:**
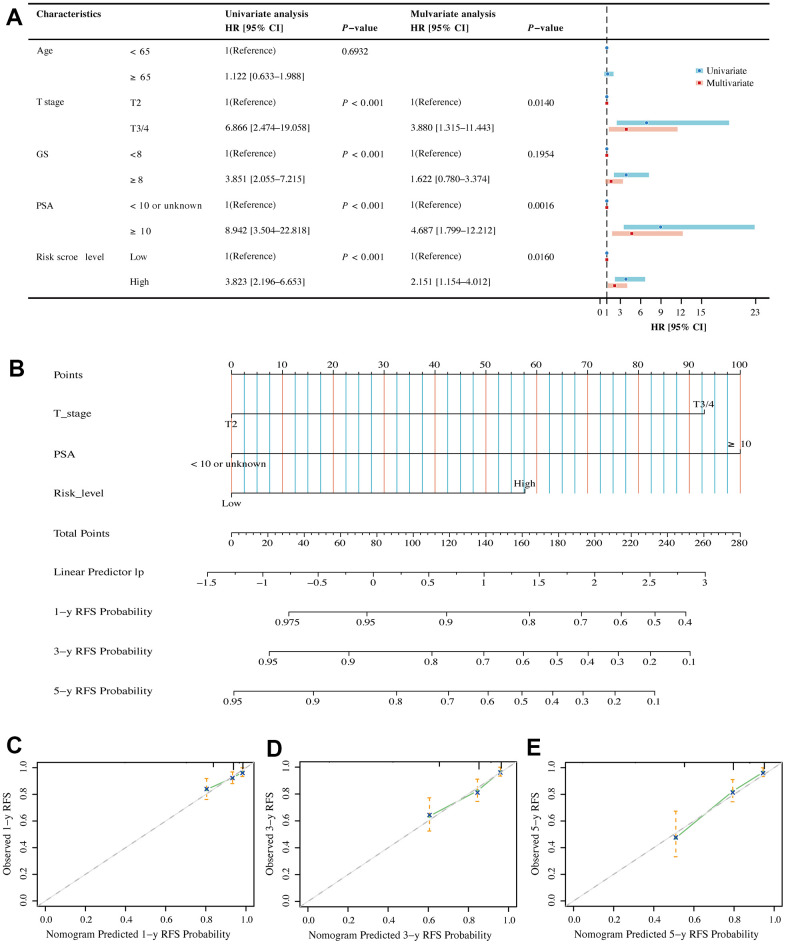
**Assessment of independent prognostic value of BMRM and construction of a nomogram in PCa patients.** (**A**) Univariate and multivariate Cox regression analysis of BMRS groupings and clinicopathological parameters in the TCGA training cohort. (**B**) Prediction of 1-, 3- and 5-year RFS for patients in the TCGA-PRAD dataset using the nomogram constructed by BMRS combined with clinicopathological parameters. (**C**–**E**) Calibration curves used to describe the agreement between the 1-, 3- and 5-year RFS predicted by the nomogram and the actual RFS of PCa patients.

Univariate and multivariate Cox regression analyses were performed in the TCGA dataset to compare the correlation between clinical information (including age, T stage, PSA and Gleason score) and BMRS in PCa patients. Forest plots indicated that BMRS may act as an independent factor for RFS in PCa (Figure 7A). Moreover, the same results were observed in the forest plots of the MSKCC external validation set (Supplementary Figure 1). We then integrated clinical information from the TCGA dataset to generate a nomogram including BMRS to predict RFS at 1, 3, and 5 years for PCa patients (Figure 7B). As expected, the calibration curves for the nomogram showed a high degree of agreement between the predicted and the actual RFS values of the patients (Figure 7C–7E). These results suggest that BMRM based on five key genes can effectively predict RFS in PCa patients.

### Exploring immune characteristics and sensitive drugs based on BMRS grouping

**Figure 8:**
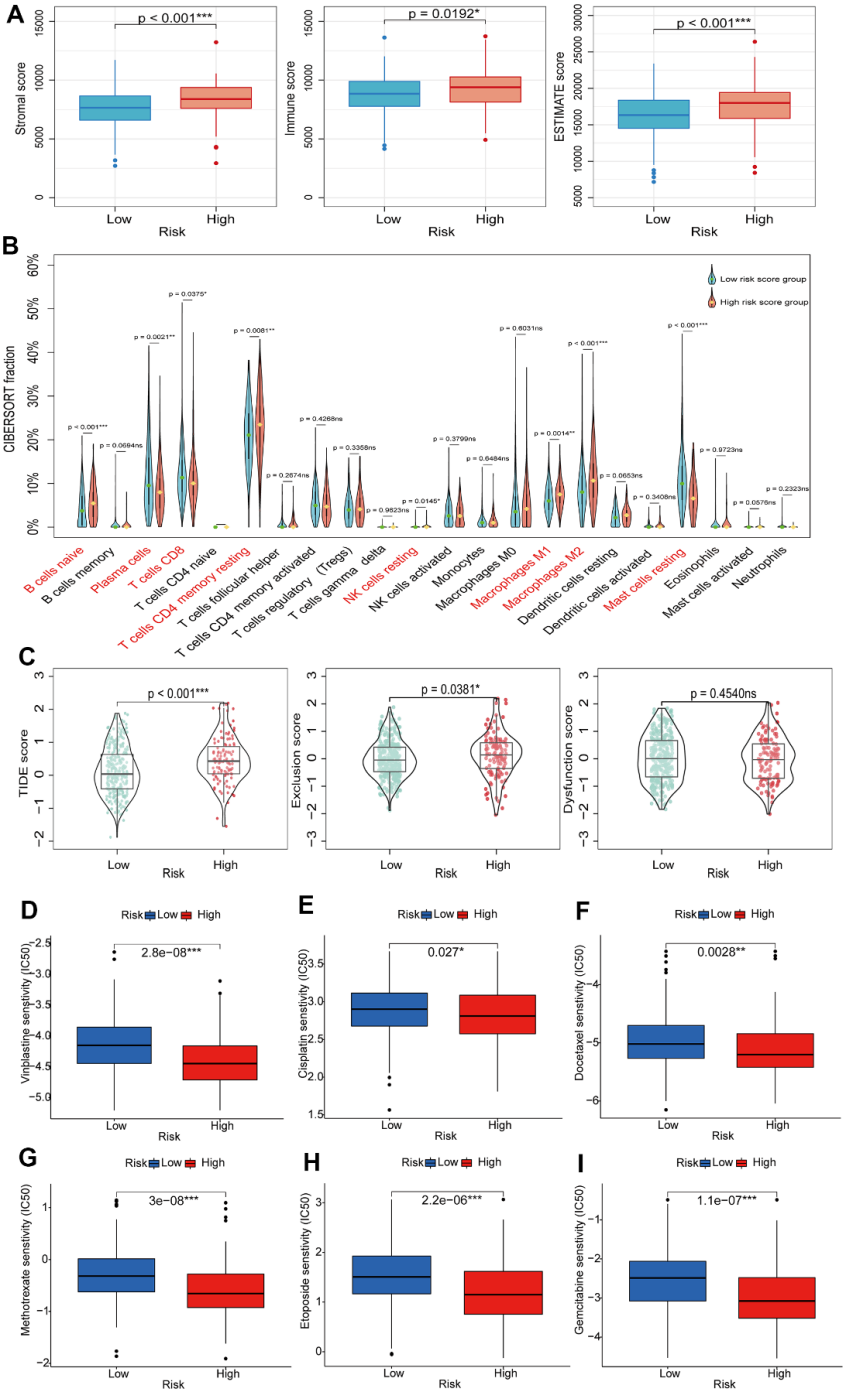
**Exploring the relationship between BMRS and TIME, immunotherapy and chemotherapy.** (**A**) Comparison of stromal score, immune score and ESTIMATE score between the high-risk and low-risk groups. (**B**) Differences in tumor immune cell infiltration between the high-risk and low-risk groups. (**C**) TIDE scores between high-risk and low-risk groups. (**D**–**I**) Association between BMRS and IC50 of chemotherapeutic agents in PCa patients, including Vinblastine, Cisplatin, Docetaxel, Methotrexate, Etoposide and Gemcitabine. ns: *p* ≥ 0.05, * *p* < 0.05, ** *p* < 0.01, *** *p* < 0.001. TIDE, Tumor Immune Dysfunction and Exclusion. ICIs, immune checkpoint inhibitors. TIME, tumor immune microenvironment.

The TIME is a highly heterogeneous ecosystem that typically contains a collection of tumor cell populations, immune cells, and tissue-specific resident and recruited stromal cell types. The study of TIME has provided theoretical support for the development of tumor immunotherapy [29]. Firstly, we explored the relationship between BMRS and TIME. The stromal score, immune score, and ESTIMATE score were all higher in the high-risk group than in the low-risk group (Figure 8A). Next, we compared the relative proportions of 22 immune cell types in samples from the two risk groups in the TCGA training set using CIBERSORT. Compared to the low-risk group, the high-risk group had a higher proportion of most tumor immune cells (Figure 8B), including initial B cells (*p* < 0.001), dormant CD4 memory cells (*p* = 0.0081), dormant natural killer cells (*p* = 0.145), M1-type macrophages (*p* = 0.0014), and M2-type macrophages (*p* < 0.001). ICIs are profoundly changing the therapeutic outlook for many cancers. Therefore, we screened for some classical ICIs and analyzed the correlation between BMRS and ICIs in PCa patients. The scatter plot results showed statistically significant expression of most immune checkpoint genes in both risk groups (Supplementary Figure 2). Interestingly, there was a significant positive correlation between BMRS and these seven immune checkpoints (BTLA, CD27, CTLA4, GPR65, HAVCR2, TIGIT and VSIG4; *p* < 0.001) and a significant negative correlation with CD38 (*p* < 0.001). In addition, we assessed the possible immunotherapy benefit between high- and low-risk individuals by TIDE score. It was shown that low-risk patients had significantly lower TIDE scores and immune escape capacity compared to high-risk patients, indicating a greater likelihood of benefiting from immunotherapy (Figure 8C). Finally, we also investigated the therapeutic response of PCa patients in both risk groups to chemotherapeutic agents. The results showed that patients in the high-risk group had a lower IC50, implying higher treatment sensitivity and response rates to Vinblastine, Cisplatin, Methotrexate, Docetaxel, Etoposide and Gemcitabine (Figure 8D–8I).

### Potentially related biological mechanisms based on BMRS grouping

**Figure 9:**
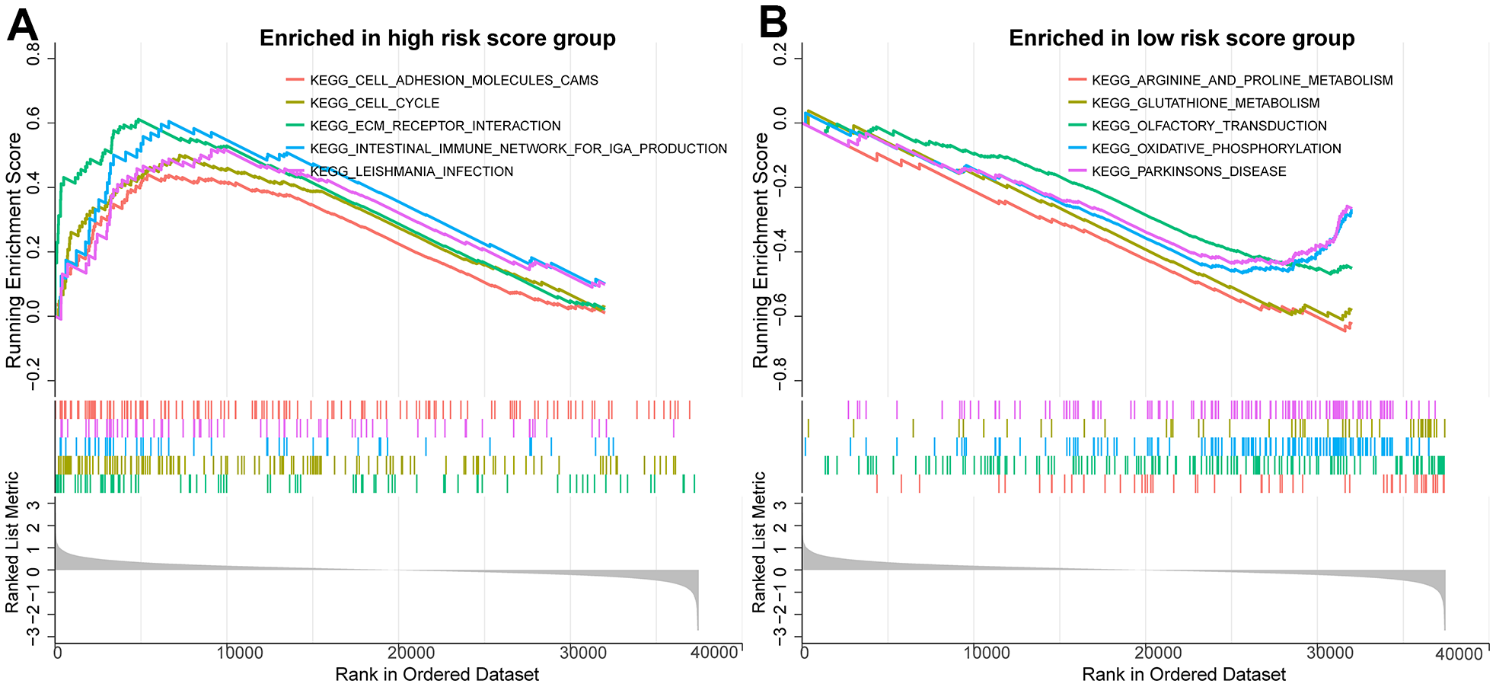
**Potential biological mechanisms for prognostic analysis of BMRM.** (**A**) GSEA analysis reveals the enriched KEGG pathways in the high-risk group. (**B**) GSEA analysis reveals the enriched KEGG pathways in the low-risk group.

To further investigate the biological mechanisms potentially associated with the PCa prognostic model, we performed a gene set registration analysis (GSEA) of DEGs in the high-risk and low-risk groups of the TCGA training set. As shown in Figure 9A, 9B, we found that cell adhesion molecules, cell cycle and ECM receptor interactions were more active in the high-risk group, and the above three biological process features were closely related to tumorigenesis and progression. Arginine and proline metabolism, glutathione metabolism and oxidative phosphorylation were enriched in the low-risk group.

### THBS2 promotes proliferation and migration in PCa cells

**Figure 10:**
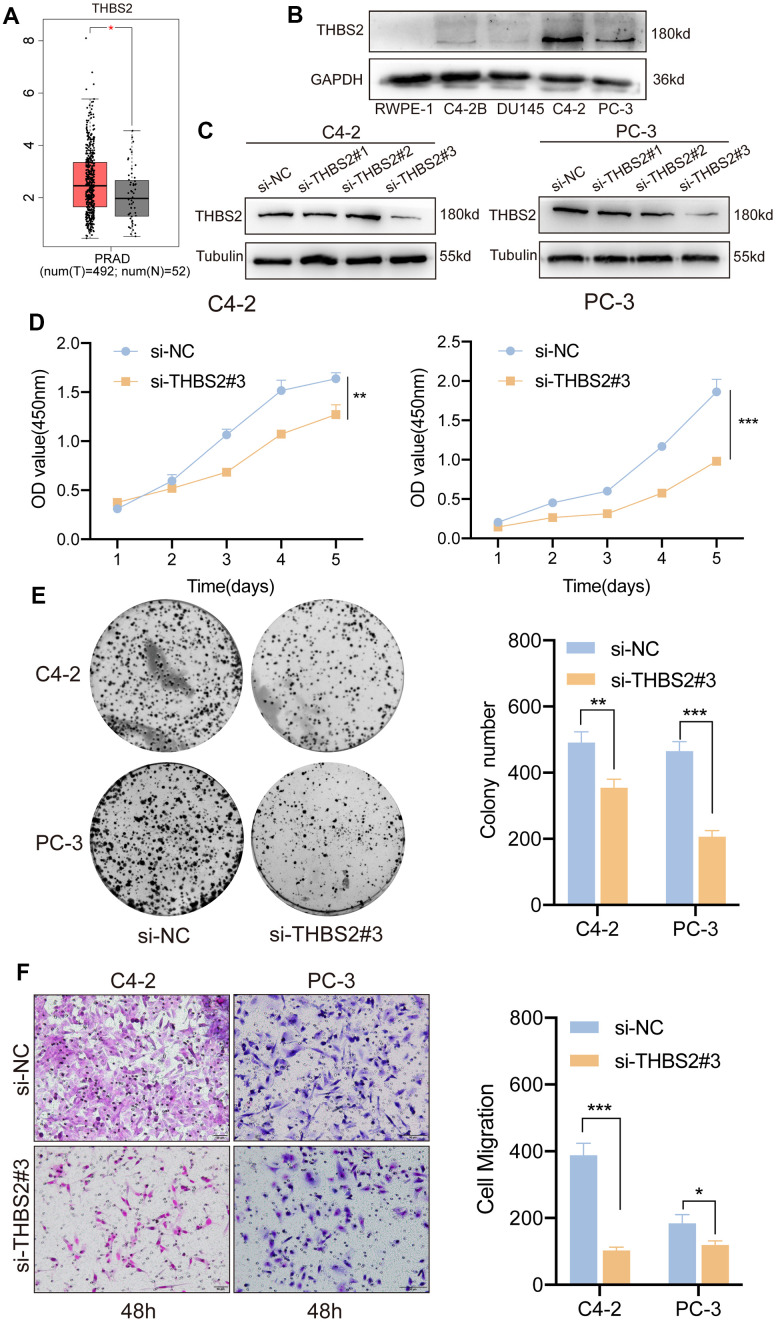
**Inhibition of THBS2 affects the proliferation and migration of PCa cells *in vitro*.** (**A**) The expression of THBS2 in TCGA PCa tumor tissues (n = 492) and the normal (n = 52). (**B**) Protein expression of THBS2 in the normal prostate epithelial cell line (RWPE-1) and PCa cell lines (C4-2B, DU145, C4-2 and PC-3). (**C**) Western blotting showing THBS2 siRNA knockdown compared to control siRNA treatment. (**D**) CCK-8 assay showed that THBS2 knockdown inhibited C4-2 and PC-3 cell proliferation. (**E**) The colony formation assay showed that THBS2 knockdown inhibited C4-2 and PC-3 colony formation. The graph on the right shows the colony numbers from 3 independent experiments. (**F**) Transwell assays showed that THBS2 knockdown inhibited C4-2 and PC-3 cell migration (Scale bar, 50 μm). The graph on the right shows the migrated cells from 3 independent experiments. All data are presented as the mean ± SD, *p < 0.05, **p < 0.01, ***p < 0.001.

Univariate and multifactorial Cox regression analyses confirmed that a high level of THBS2 expression was an independent adverse prognostic factor in PCa. However, its biological function in PCa remains unclear. TCGA database analysis revealed high THBS2 expression in PCa tissue compared to normal prostate tissue (Figure 10A). In addition, we also compared THBS2 expression levels between PCa cell lines (C4-2, C4-2B, DU145, and PC-3) and the normal prostate epithelial cell line (RWPE-1) by Western blotting analysis. The results showed that THBS2 was significantly upregulated in PCa cell lines, especially in C42 and PC-3 cells compared to RWPE-1 (Figure 10B), further demonstrating the upregulated expression of THBS2 in PCa. We used three siRNAs (si-THBS21#1, si-THBS2#2, and si-THBS2#3) to transiently knockdown THSB2 in two PCa cell lines C4-2 and PC-3. To evaluate the knockdown efficacy of THBS2, Western blotting showed that THBS2 expression was significantly reduced in C4-2 and PC-3 cells after transfection with si-THBS2#3 (Figure 10C). We chose si-THBS2#3 for the subsequent experiments due to it having the best efficiency of interference. Experiments have shown that the knockdown of THBS2 can inhibit the proliferation and colony forming abilities of C4-2 and PC-3 (Figure 10D, 10E). In addition, according to the migration results of Transwell, the silencing of THBS2 can significantly inhibit the migration of C4-2 and PC-3 (Figure 10F). Collectively, these results demonstrate that THBS2 promotes PCa cell growth and migration.

## DISCUSSION

Tumor metastasis is the most common cause of cancer-related death [30]. The basement membrane acts as a resilient ECM and once the structure and function of the basement membrane are altered, cancer cells may break through the basement membrane and spread or metastasize [31, 32]. A growing number of studies have shown that the basement membrane plays an important role in the progression of PCa, influencing the migration and invasion of tumor cells [33, 34]. Therefore, it is imperative to seek biomarkers to construct risk prediction models that can predict early recurrence in PCa patients.

We downloaded transcriptome sequencing data and clinical information about PCa patients from the TCGA database as a training set. The five BMGs (THSB2, COL4A6, MMP21, MMP26 and SDC1) were identified as independent prognostic factors for RFS in PCa patients using stepwise multiple regression analysis. Previous studies have shown that BMGs are associated with PCa pathophysiology. He et al. found that THBS2 is a metastasis-specific biomarker and poor survival key regulator in human colon cancer [35]. Downregulation of collagen COL4A6 is associated with PCa progression and metastasis [36]. Recent studies have shown that soluble syndecan-1 (SDC1) serum level is an independent pre-operative predictor of cancer-specific survival in PCa, and SDC1 is associated with more aggressive tumors and a worse prognosis [37]. In addition, matrix metalloproteinases (MMPs) are a class of zinc-dependent endoproteases responsible for tissue remodeling and degradation of ECM proteins [38]. There are at least 23 matrix metalloproteinases expressed in humans, and MMP21 and MMP26 have been reported to play an important role in the progression of several cancers, including PCa [39, 40].

We further constructed BMRM from five carefully selected BMGs and classified PCa patients into high- and low-risk groups according to the risk model. In the MSKCC cohort validation set, the ROC curves demonstrated the high sensitivity and specificity of our risk model in predicting 1-year, 3-year and 5-year RFS in PCa patients with different risk scores. In addition, we have combined the risk scores with different clinical characteristics to produce a nomogram with good predictive power, improving clinical applicability. Statistically significant differences in T-stage, Gleason score, and PSA were found between the different risk groups, indicating a greater likelihood of tumor progression and recurrence in PCa patients in the higher risk groups.

To further investigate the biological mechanisms potentially relevant to the prognostic risk assessment of BMRM, we performed a GSEA of DEGs between the high-risk and low-risk groups of the TCGA training set. We found that cell adhesion molecules, cell cycle and ECM receptor interaction pathways were more significantly enriched in the high-risk group. Among these, cell adhesion and cell cycle are both common signaling pathways in cancer research, mediating tumor proliferation, metastasis and treatment resistance [41]. Tumor cells interact with the ECM through receptors such as fibronectins and integrins, thereby transducing multiple signals to regulate critical cell differentiation, cell proliferation and migration [42].

TMB indicates the number of mutations per megabase (Mut/Mb) in DNA sequenced in cancers [43]. Studies have shown that TMB is considered a reliable predictive biomarker of efficacy in cancer patients treated with ICIs [44, 45]. In the TCGA cohort, we found a significant correlation between TMB and BMRS. High-risk patients had a higher TMB and a poorer RFS, while low-risk patients had a lower TMB and a better RFS. In addition, the proportion of TP53 mutations was significantly higher in the high-risk group compared to the mutation profile of patients in the low-risk group. The rate of TP53 mutations was consistent with previous reports that TP53 mutations were frequently detected in patients with high-risk PCa. Moreover, mutations in TP53 appeared more frequently in metastatic castration-resistant prostate cancer (mCRPC) compared with primary tumor [46, 47]. Furthermore, the TP53 gene is an important oncogene, and its mutation frequency is associated with poor outcomes and treatment resistance in PCa patients [48, 49].

Currently, tumor immunotherapy has significantly advanced the landscape of cancer treatment [50]. Unlike other malignancies, PCa as an immunologically “cold” tumor has an immunosuppressive microenvironment and therefore the development of immunotherapy has lagged behind [51, 52]. In the future, a comprehensive and accurate assessment of the immune microenvironment of PCa and exploration of the underlying mechanisms of immunosuppression and immune escape will be an effective and important means of advancing the pace of immunotherapy. Therefore, we used the CIBERSORT algorithm to assess the relative proportions of 22 immune cell types in PCa. Depletion of CD8+ T-cells is detrimental to the anti-tumor immune response and is associated with a worse prognosis [53]. In addition, tumor-associated macrophages (TAMs), an important component of the immune microenvironment, mainly M2-type TAMs promote tumor growth and metastasis [27]. Consistent with our study, the high-risk group in this model had a lower abundance of CD8+ T-cells and a higher abundance of M2-type TAMs, suggesting that this BMRM provides clues to the study of immunotherapy and has implications for immunotherapy decisions in PCa patients.

ICIs have brought survival benefits for some oncology patients, but response rates are low in patients with mCRPC [54, 55]. We performed a correlation analysis of risk scores with selected ICIs, and the expression of BTLA, CD27, CTLA4, GPR65, HAVCR2, TIGIT and VSIG4 was upregulated in the high-risk group, suggesting that these new immune checkpoints may serve as potential candidate immunotherapy targets for PCa. TIDE scores have been reported to be negatively correlated with responsiveness to anti-PD-1 and anti-CTLA-4 therapy [56]. In our study, the higher TIDE score in the high-risk group implied that the efficacy of ICIs therapy might be worse. In summary, patients in the low-risk group were more likely to benefit from immunotherapy. However, the role of single immunotherapy in advanced metastatic PCa is very limited, and therefore targeted therapy or chemotherapy combined with immunotherapy are promising treatment options for the future [51, 57]. Next, we explored the sensitivity of different BMRS groups to specific chemotherapeutic agents (Vinblastine, Cisplatin, Methotrexate, Docetaxel, Etoposide and Gemcitabine), and the results revealed a higher response rate to these chemotherapeutic agents in the higher risk groups.

This study explores the potential role of BMGs in the biochemical recurrence and therapeutic assessment of PCa. Constructing a BMRM by screening reliable BMGs may provide clues for clinical treatment decisions and patient prognostic assessment in PCa. However, our BMRM still has some limitations. First, although we have explored the risk score of our model in the TCGA training set and MSKCC validation set, validation of differential gene expression in an expanded sample size may further improve the confidence of the risk score. Secondly, this study confirms the good predictive value of the prognostic features of BMGs, but *in vivo* and *in vitro* experiments are still needed to reveal the role of BMGs in the development and progression of PCa. Thirdly, more clinical data and prospective studies are still needed to validate the clinical value of the prognostic features of carefully selected BMGs.

In conclusion, we developed a new BMGs-based prognostic model to determine the prognostic value of BMGs in PCa. Furthermore, BMRS correlates with TIME characteristics, ICIs, and chemotherapeutic drug sensitivity, which may provide potential evidence for patient treatment selection. In addition, the key gene THBS2 of BMGs may be an important link in the genesis and progression of PCa. This study provides a novel perspective to assess the prognosis of PCa patients and provides clues for the selection of future personalized treatment regimens.

## Supplementary Material

Supplementary Figures

Supplementary Tables
