# Supplementary material for: Identification of a basement membrane gene signature for predicting prognosis and estimating the tumor immune microenvironment in prostate cancer
Source: Aging (Albany NY). 2024 Jan 17;16(2):1581–604. doi: 10.18632/aging.205445 (PMC10866409; doi:10.18632/aging.205445)
Supplement: Supplementary Figures [file aging-16-205445-s001.pdf]

SUPPLEMENTARY FIGURES

Characteristics		Univariate analysis HR [95% CI]	*P*-value	Mulvariate analysis HR [95% CI]	*P*-value
Age	< 65	1 (Reference)	0.3971		
	≥ 65	1.429 [0.625–3.267]			
T stage	T2	1 (Reference)	$p < 0.001$	1 (Reference)	$p < 0.001$
	T3/4	5.234 [2.565–10.681]		3.938 [1.883–8.237]	
GS	< 8	1 (Reference)	$p < 0.001$	1 (Reference)	0.0064
	≥ 8	4.459 [2.082–9.551]		2.984 [1.361–6.546]	
PSA	< 10 or unknown	1 (Reference)	0.0592		
	≥10	2.026 [0.973–4.220]			
Risk score level	Low	1 (Reference)	$p < 0.001$	1 (Reference)	0.0130
	High	3.961 [2.053–7.642]		2.384 [1.201–4.733]	

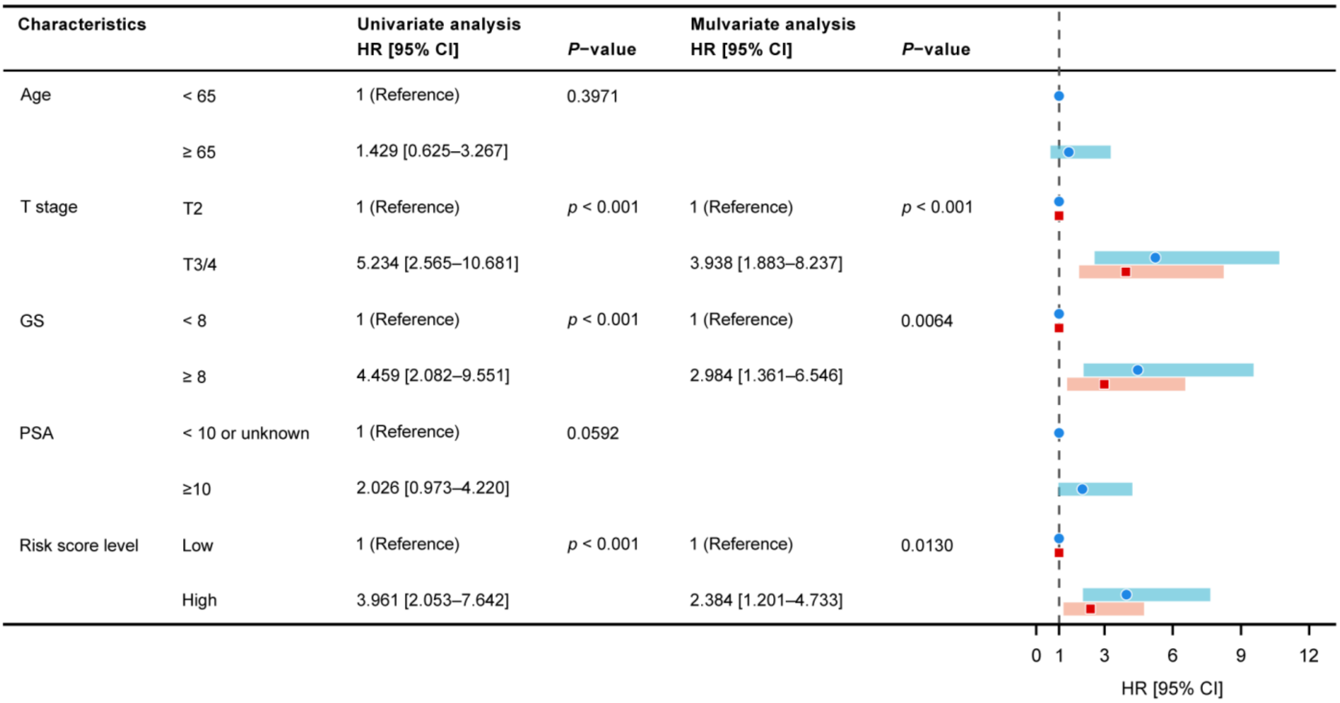

Supplementary Figure 1. The forest maps show the results of univariate and multivariate Cox regression analyses in the MSKCC validation set.

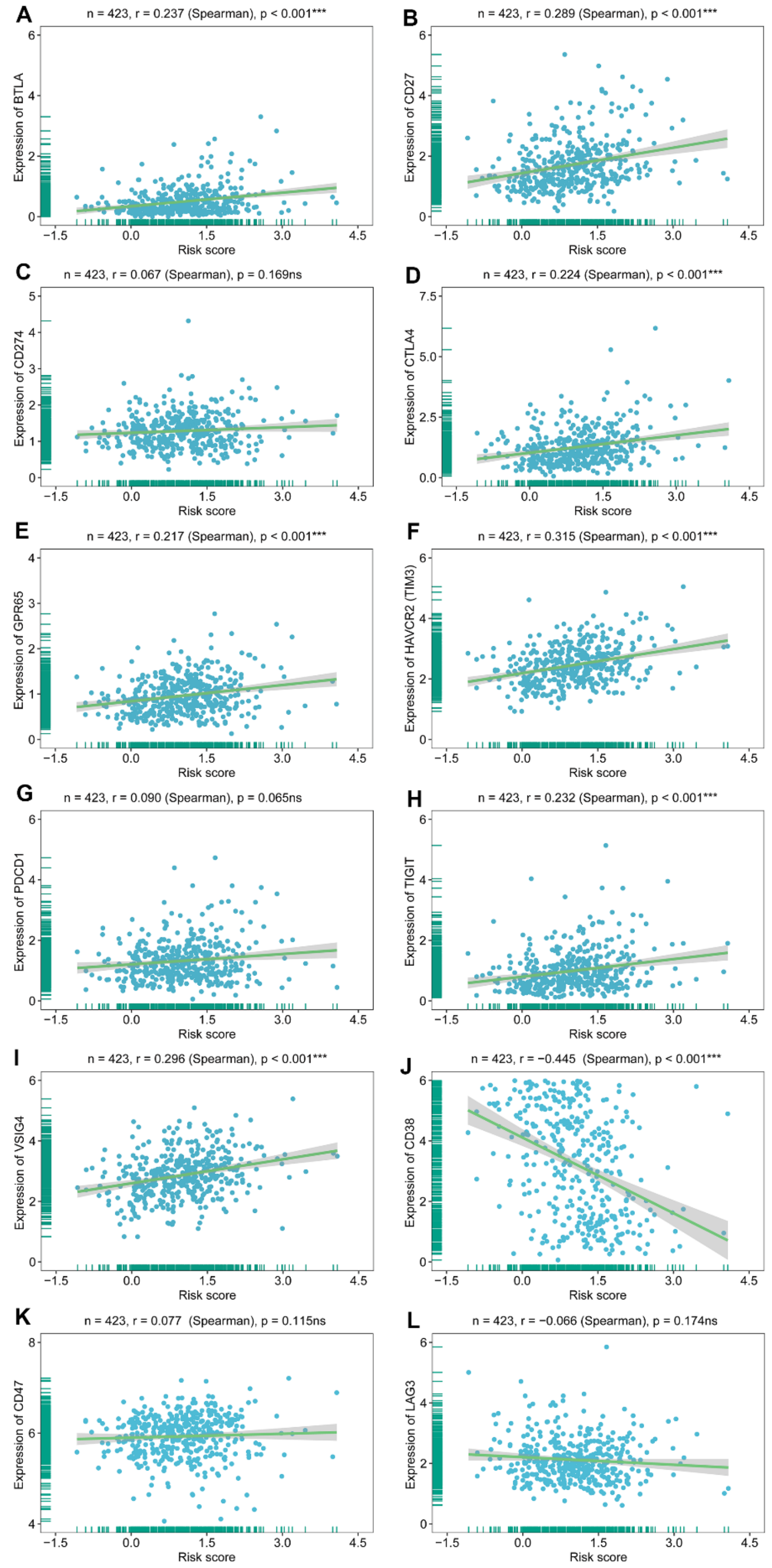

Supplementary Figure 2. Correlation analysis between BMRS and immune checkpoints. (**A**–**L**) The scatter plot results show statistically significant expression of most immune checkpoint genes in both risk groups. There was a significant positive correlation between BMRS and these seven immune checkpoints (BTLA, CD27, CTLA4, GPR65, HAVCR2, TIGIT and VSIG4) and a significant negative correlation with CD38. ns: $p \geq 0.05$, *$p < 0.05$, **$p < 0.01$, ***$p < 0.001$.
