# Supplementary material for: Identification of a basement membrane gene signature for predicting prognosis and estimating the tumor immune microenvironment in prostate cancer
Source: Aging (Albany NY). 2024 Jan 17;16(2):1581–604. doi: 10.18632/aging.205445 (PMC10866409; doi:10.18632/aging.205445)
Supplement: Supplementary Tables [file aging-16-205445-s002.pdf]

SUPPLEMENTARY TABLES

Supplementary Table 1. A list of 224 basement membrane genes.

Basement membrane genes (BMGs)							
ACAN	COL5A1	FREM1	MMP2	ACHE	FBN3	LOXL4	ROBO1
ADAM10	COL6A1	FREM2	MMP21	ADAMTS1	FMOD	LUM	SDC1
ADAM17	COL6A2	GPC3	MPZL2	ADAMTS14	FREM3	MATN1	SDC4
ADAM9	COL6A3	GPC4	MUSK	ADAMTS15	GPC1	MATN2	SEMA3B
ADAMTS10	COL7A1	GPC6	NTN1	ADAMTS16	GPC2	MATN4	SLIT1
ADAMTS13	COL8A2	HMCN1	P3H1	ADAMTS19	GPC5	MEGF6	SLIT2
ADAMTS17	COL9A1	HSPG2	P3H2	ADAMTS20	HAPLN1	MEGF9	SLIT3
ADAMTS18	COL9A2	ITGA2B	PTPRF	ADAMTS4	HAPLN2	MEP1A	SPARCL1
ADAMTS2	COL9A3	ITGA3	PXDN	ADAMTS5	HMCN2	MEP1B	SPOCK1
ADAMTS3	COLQ	ITGA6	ROBO2	ADAMTS6	ISLR	MMP17	SPOCK2
AGRN	CST3	ITGA7	ROBO3	ADAMTS7	ITGA1	MMP26	SPOCK3
AMELX	CTSA	ITGA8	ROBO4	ADAMTS8	ITGA10	MMP7	SPON1
AMTN	CTSB	ITGB2	RPSA	ADAMTS9	ITGA2	MMRN2	SPON2
ANG	CTSD	ITGB3	SERPINF1	BCAN	ITGA4	NELL1	TENM1
BGN	DAG1	ITGB4	SMC3	CCDC80	ITGA5	NELL2	TENM2
CD151	DCC	ITGB6	SMOC1	CD44	ITGA9	NID1	THBS1
CERT1	DCN	LAMA1	SMOC2	COL14A1	ITGAM	NID2	THBS2
COL12A1	DDR2	LAMA2	SPARC	COL15A1	ITGAV	NPNT	THBS4
COL13A1	ECM1	LAMA3	TENM3	COL28A1	ITGAX	NTN4	TIMP1
COL17A1	EFEMP1	LAMA4	TENM4	COL8A1	ITGB1	OGN	TIMP2
COL18A1	EFEMP2	LAMB1	TGFB1	CSPG4	ITGB5	OPTC	TINAG
COL2A1	FBLN1	LAMB2	TGFB2	DDR1	ITGB7	PAPLN	TINAGL1
COL4A1	FBLN5	LAMB3	TGFBI	EGFL6	ITGB8	PHF13	UNC5A
COL4A2	FBN1	LAMC2	TIMP3	EGFLAM	LAD1	PODN	UNC5B
COL4A3	FBN2	LAMC3	TLL1	EVA1A	LAMA5	POSTN	UNC5C
COL4A4	FGF9	LOXL1	TNC	EVA1B	LAMB4	PTN	UNC5D
COL4A5	FN1	MMP1	USH2A	EVA1C	LAMC1	PXDNL	VTN
COL4A6	FRAS1	MMP14	VCAN	FBLN2	LOXL2	RECK	VWA1

Supplementary Table 2. The clinicopathological characteristics of patients in the TCGA-PRAD cohort and MSKCC cohort.

Characteristics	TCGA-PRAD (n=423)	MSKCC (n=140)
Age(year)		
<65	283	117
≥65	140	23
T stage		
T1/2	154	86
T3/4	269	54
Gleason score		
<8	241	127
≥8	182	13
PSA (ng/mL)		
<10 or unknown	409	116
≥10	14	24
Recurrent events	52	36

Supplementary Table 3. The siRNA oligos used for knock-down of THBS2.

Name	Target sequence	Note
si-THBS2#1	GTGGCACATTCTACGTAAA	siRNA Knock-down
si-THBS2#2	GGACCTATCTATGACCAAA	siRNA Knock-down
si-THBS2#3	GGAACATTGGCTGGAAGGA	siRNA Knock-down
